# Microarray screening of Guillain-Barré syndrome sera for antibodies to glycolipid complexes

**DOI:** 10.1212/NXI.0000000000000284

**Published:** 2016-09-28

**Authors:** Susan K. Halstead, Gabriela Kalna, Mohammad B. Islam, Israt Jahan, Quazi D. Mohammad, Bart C. Jacobs, Hubert P. Endtz, Zhahirul Islam, Hugh J. Willison

**Affiliations:** From the Institute of Infection, Immunity and Inflammation (S.K.H., H.J.W.), College of Medical, Veterinary and Life Sciences, University of Glasgow; The Beatson Institute for Cancer Research (G.K.), Glasgow, UK; Laboratory Sciences and Services Division (M.B.I., I.J., Q.D.M., Z.I.), International Centre for Diarrheal Disease Research; Shaheed Tajuddin Ahmed Sarani (M.B.I., I.J., Q.D.M., Z.I.), Mohakhali, Dhaka, Bangladesh; Departments of Immunology and Neurology (B.C.J.) and Medical Microbiology and Infectious Diseases (H.P.E.), Erasmus MC, University Medical Center Rotterdam, Rotterdam, the Netherlands; and Fondation Mérieux (H.P.E.), Lyon, France.

## Abstract

**Objective:**

To characterize the patterns of autoantibodies to glycolipid complexes in a large cohort of Guillain-Barré syndrome (GBS) and control samples collected in Bangladesh using a newly developed microarray technique.

**Methods:**

Twelve commonly studied glycolipids and lipids, plus their 66 possible heteromeric complexes, totaling 78 antigens, were applied to polyvinylidene fluoride–coated slides using a microarray printer. Arrays were probed with 266 GBS and 579 control sera (2 μL per serum, diluted 1/50) and bound immunoglobulin G detected with secondary antibody. Scanned arrays were subjected to statistical analyses.

**Results:**

Measuring antibodies to single targets was 9% less sensitive than to heteromeric complex targets (49.2% vs 58.3%) without significantly affecting specificity (83.9%–85.0%). The optimal screening protocol for GBS sera comprised a panel of 10 glycolipids (4 single glycolipids GM1, GA1, GD1a, GQ1b, and their 6 heteromeric complexes), resulting in an overall assay sensitivity of 64.3% and specificity of 77.1%. Notable heteromeric targets were GM1:GD1a, GM1:GQ1b, and GA1:GD1a, in which exclusive binding to the complex was observed.

**Conclusions:**

Rationalizing the screening protocol to capture the enormous diversity of glycolipid complexes can be achieved by miniaturizing the screening platform to a microarray platform, and applying simple bioinformatics to determine optimal sensitivity and specificity of the targets. Glycolipid complexes are an important category of glycolipid antigens in autoimmune neuropathy cases that require specific analytical and bioinformatics methods for optimal detection.

Autoantibody binding to glycolipids that act as antigens in patients with autoimmune neuropathy is heavily influenced by the topographic orientation of the carbohydrate head group within living peripheral nerve tissue, and also within an immunoassay microenvironment.^1^ Thus, clusters of different lipids can interact to form complex molecular shapes capable of acting as antigens that are not detectable when assaying for individual glycolipid reactivities.^2,3^ Equally, some antibody-binding sites on glycolipids may be obscured when the glycolipid is part of a larger, heteromeric lipid cluster. This new category of glycolipid complex–dependent autoantibodies has recently been described as either complex enhanced or complex attenuated. Antiglycolipid antibodies whose binding is unaffected by clustering are referred to as complex independent.

Incorporating such findings into the design of screening assays for antiglycolipid antibodies adds substantial complexities to what is already a difficult assay platform to standardize, even when using single glycolipids as antigens. Thus if one considers 20 different glycolipids as targets, the number of possible heteromeric complexes in a 1:1 ratio amounts to 180. If one adds a third lipid to the cluster, or diversifies the ratios of the cluster components, combinatorial complexity rises to unmanageable proportions when using routinely established ELISA-based immunoassays.

To account for this, and allow us to screen for antibodies to highly varied glycolipid complexes in an unbiased way, we have developed a microanalytical method for assaying cohorts of sera against multiple combinatorial targets that advances our previously reported methods. In this proof of principle study, we selected 12 glycolipids or lipids, plus their 66 possible heteromeric complexes, totaling 78 antigens. We applied this assay to identify previously reported combinatorial targets in a screen of 845 Guillain-Barré syndrome (GBS) and control samples collected in Dhaka, Bangladesh. We expected that this geographical setting would provide a high proportion of axonal GBS variants and potentially a high chance of capturing antibodies to ganglioside complexes, as have been previously observed in acute motor axonal neuropathy (AMAN). Particularly in near-patient settings where early diagnosis is useful, simple biomarker testing kits carrying high sensitivity and specificity that could be derived from these more complex datasets are needed. We report the findings and identify the major clinically useful targets in this patient group.

## METHODS

### Array fabrication.

For full methods, please refer to the e-Methods at Neurology.org/nn. In brief, array platforms were produced in-house from polyvinylidene fluoride membrane adhered to glass microscope slides. Working solutions of single glycolipids were prepared at 200 μg/mL in methanol, from which heteromeric complexes were prepared. Glycolipid samples were stored at −20°C and sonicated prior to printing. Glycolipids microarray slides were produced using a Sciflexarrayer S3 microarray printer (Scienion; Berlin, Germany). A maximum of 20 slides was printed per run, each containing 16 subarrays per slide (320 arrays in total). All glycolipid targets were printed in duplicate on each array and included methanol solvent, which was printed as a negative control. Upon completion of printing, arrays were stored at 4°C until required.

### Clinical samples.

A total of 845 patients with GBS (with associated clinical data) and control group sera were collected at Dhaka Medical College Hospital, Bangladesh, between 2010 and 2013. GBS cases were enrolled according to National Institute of Neurological Disorders and Stroke criteria.^4^ Samples comprised 266 patient sera (GBS), 258 family controls (FC), and 321 other neurologic disease controls (NC). Samples were stored at −70/80°C in the Laboratory Sciences and Services Division. All patients provided written informed consent; the study was approved by the ethics committees of the International Centre for Diarrhoeal Disease Research, Dhaka, Bangladesh, and Dhaka Medical College, Bangladesh.

### Sera screening.

Nonspecific serum binding was reduced by blocking arrays in 2% bovine serum albumin (BSA)/phosphate-buffered saline (PBS). Each of the 16 subarrays per slide were isolated using a FAST frame including a 16-well incubation chamber (Maine Manufacturing, Sanford, ME) and 100 μL of each serum sample, diluted 1:50 in 1% BSA/PBS, was applied per well for 1 hour at 4°C. Samples were removed from each chamber and then washed twice in 1% BSA/PBS for 15 minutes at room temperature. Antibody binding was detected with 100 μL of 2 μg/mL Alexafluor 647 conjugated goat antihuman immunoglobulin G (IgG) (Jackson ImmunoResearch, West Grove, PA) per well for 1 hour at 4°C. The arrays were then washed twice in 1% BSA/PBS for 30 minutes, followed by twice in PBS for 5 minutes and a final 5 minutes wash in distilled water. Each serum sample was assayed in duplicate and screened twice in 2 independent assays.

### Scanning and analysis.

Arrays were scanned and quantitated using a PerkinElmer (Waltham, MA) ScanArray Express instrument. Image analysis was carried out with ProScanArray Express Easy Quant software. Each target spot was measured for median fluorescence intensity with local background median pixel intensity subtracted, and the mean value was calculated for each pair of duplicate spots. Values obtained from repeat runs were averaged and used in all calculations. Data processing was performed with Microsoft (Redmond, WA) Excel. Statistical analysis was performed using censReg R, GraphPad prism, MedCalc, and MeV software. Array data were compared with previously determined ELISA data for GM1 positivity in 261/266 of the GBS cases and showed 88.9% agreement (positive or negative).

## RESULTS

### Patient and control sample demographics.

Suspected GBS cases recruited for this study (n = 299) had the diagnosis of GBS confirmed on subsequent clinical evaluation in 266 patients. A total of 183 were male (68.8%) and 83 were female (31.2%). The median/mean age of patients with GBS was 28/30 years (range 1–75). Electrophysiologic examination at one time point after clinical onset was performed on 192 patients (72.2%) and classified as follows: acute inflammatory demyelinating polyneuropathy (AIDP), 58 cases (30.2%); AMAN, 102 cases (53.1%); unclassified GBS, 32 cases (16.7%). Of the remaining 74 patients who did not undergo electrophysiologic examination, all but one patient (who was classified as AIDP) were categorized as unclassified GBS. Patients reported the following preceding symptoms (either in isolation or in combination with other symptoms): diarrhea, 130 cases (48.9%); respiratory infection, 59 cases (22.2%); fever, 34 cases (12.8%); chicken pox, 7 cases (2.6%); vaccination, 6 cases (2.3%); other, 21 cases (7.9%). No preceding symptoms were reported in 32 cases (12.0%). There were 35 deaths (13.2%) and 58 patients (21.8%) required ventilation at some point during treatment. For the family and neurologic control demographic and preceding symptom data, see the e-Methods.

### Antiglycolipid antibody screen.

**Figure 1:**
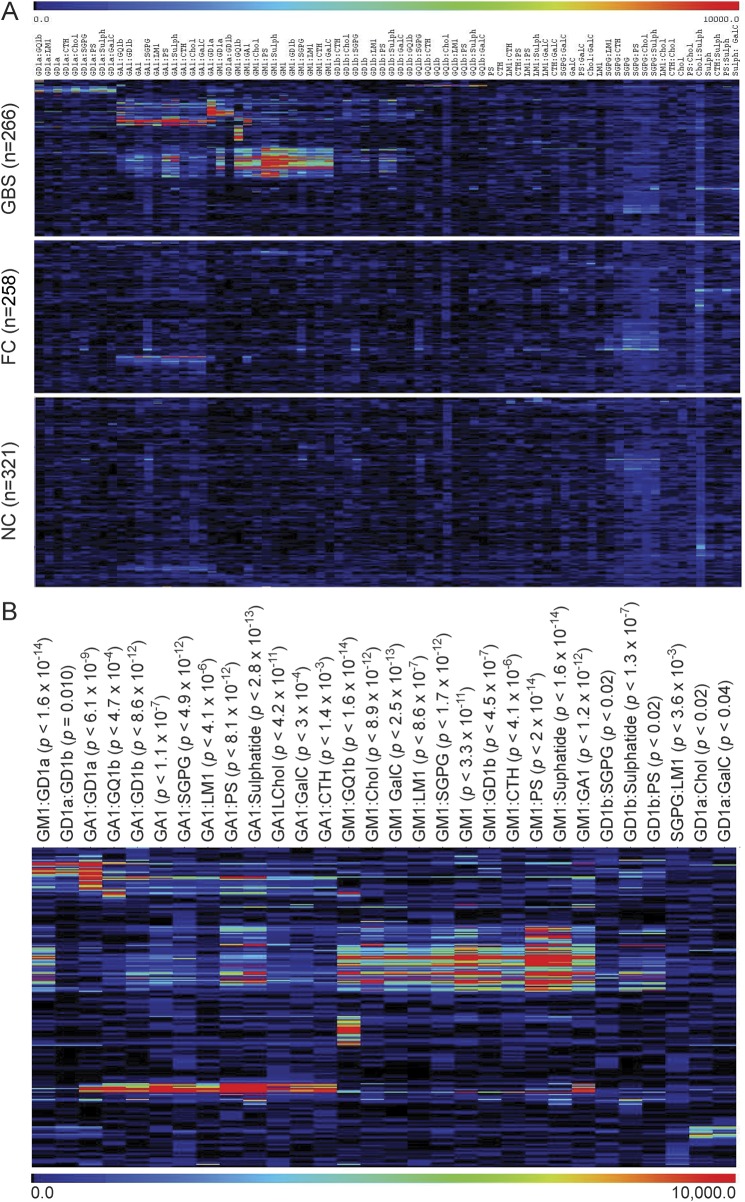
Heat map of antibody binding patterns to glycolipid targets in Guillain-Barré syndrome (GBS) cases and controls (A) Patient (GBS) and control sera (family controls [FC] and neurologic disease controls [NC]) were screened against a panel of 78 single and heteromeric glycolipid targets on glycolipid microarray. Mean fluorescent intensity values were calculated and graphically presented as a heat map. The rainbow scale indicates the intensity of the antibody binding, in which blue is weak and red is strong antibody binding. Pearson hierarchical clustering was employed to group samples with similar antigen binding patterns. (B) Heat map presentation of GBS samples for the 30 glycolipid targets identified as having significantly different binding intensities between GBS and FC or NC groups (*p* < 0.05).

The mean intensity values were calculated from 4 intensity measurements per target for each serum sample. These 78 targets are visually represented as a heat map employing Pearson cluster coefficient and displayed as 3 groups: GBS (n = 266), FC (n = 258), and NC (n = 321), with antibody binding intensity portrayed in a rainbow scale, red being high binding and blue being low binding (figure 1A). As expected for a GBS population in which multiple targets are known to be present, no single antigen binding pattern was dominant throughout the cohort examined. Instead, 30 targets were found with significantly increased (*p* < 0.05) binding intensities in GBS as compared independently with both FC and NC groups. These data on 30 significant targets are also displayed solely for the GBS group for simplicity (figure 1B). Twenty-eight of the targets in the GBS group were heteromeric complexes and 2 (GA1 and GM1) were single glycolipids. While these 30 targets are highly significantly different between disease and control groups and may be clinically useful when multiple tests are performed, screening for one antigen or antigen complex cannot be used to determine a measure of clinical usefulness by receiver operating characteristic (ROC) analysis, due to the low sensitivity of individual targets. Indeed, as expected, when subjected to ROC analysis, no targets reached area under the curve ≥0.75 (the minimal level considered to be the gold standard for a clinically useful biomarker^5^) when compared with combined (family and neurologic) control groups.

Certain targets (containing SGPG, cholesterol, and GA1) returned higher than average signals across both disease and control groups, indicating that normal ranges need to be determined for each antigen. Optimized positivity threshold values were calculated for each target antigen, defined as the 95th percentile of the combined controls. All intensity values were converted to binary data (positive/negative), determined by the optimal threshold calculated for each target, and all subsequent data are expressed in terms of sensitivity and specificity. When considering all 78 targets, a sensitivity of 82.7% and a specificity of 37.1% was achieved, indicating that 62.9% of the combined control samples had antibody binding intensity greater than the threshold for one or more of the 78 targets, despite only 5% of controls being positive for any one individual antigen target. In order to increase the specificity of the assay, we established a refined panel of target antigens.

### Anti-GM1, -GA1, -GD1a, and -GQ1b antibodies.

**Figure 2:**
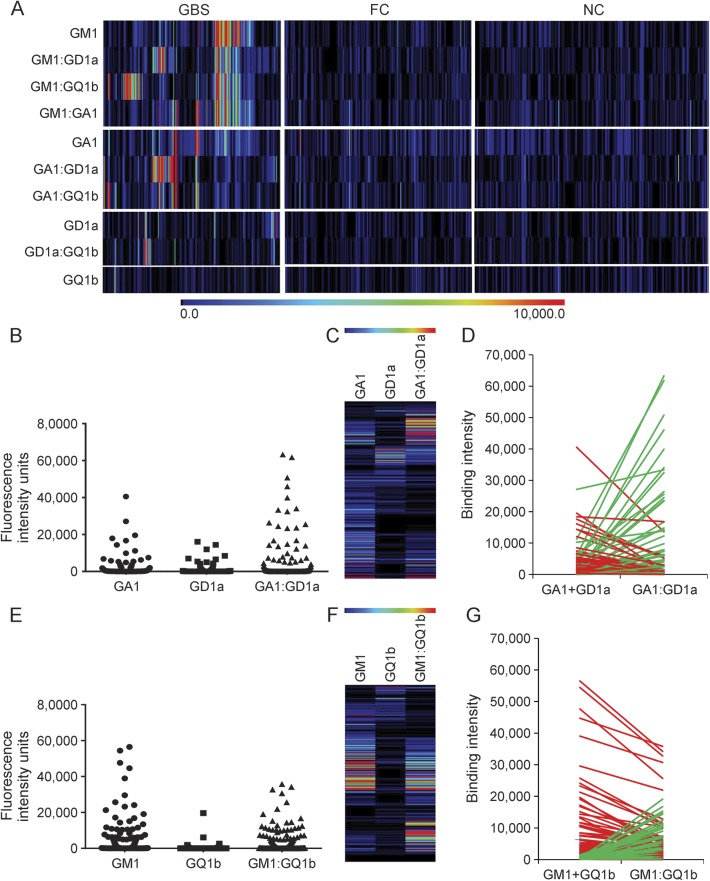
Subanalysis of 10 major antiglycolipid antibody targets in Guillain-Barré syndrome (GBS) cases (A) Heat map presentation of patient (GBS) and control sera (family controls [FC] and neurologic disease controls [NC]) reactivity with GM1, GD1a, GQ1b, and GA1 as single antigens and their 6 possible heteromeric complexes. When selecting this small panel of 10 targets as serum biomarkers of GBS in this clinical cohort, the combined sensitivity is 64.3% and the specificity is 77.1%. (B) Dot plot presentation of GBS (n = 266) antibody binding intensities for single GA1 and GD1a and their heteromeric complex, GA1:GD1a. (C) Heat map presentation of patient binding intensities. Each row represents a single patient, with binding intensity to each target represented using the rainbow scale. (D) Line graph compares the binding intensity for complex GA1:GD1a with the sum of the single antigens (GA1 and GD1a). Green lines indicates complex enhancement, while red lines represent complex attenuation. (E) Dot plot presentation of GBS (n = 266) antibody binding intensities for single GM1 and GQ1b, and their heteromeric complex, GM1:GQ1b. (F) Heat map presentation of patient binding intensities. Each row represents a single patient, with binding to each target represented using the rainbow scale. (G) Line graph compares the binding intensity for complex GM1:GQ1b with the sum of the single antigens (GM1 and GQ1b). Green lines indicates complex enhancement, while red lines represent complex attenuation.

Focusing on 4 glycolipids that are already recognized to be targets in GBS and were prominent antigens in this screen, several common cluster patterns comprising GM1, GA1, GD1a, and GQ1b alone or in complex could be identified (figure 2A). The largest group of sera contained anti-GM1 antibodies (n = 92; sensitivity = 34.6%). Many of these also bound to GM1 when in heteromeric complex; however, in some cases, binding to GM1 was inhibited when in heteromeric complex with another glycolipid. Considering the samples in which heteromeric complexes enhanced anti-GM1 antibody binding, GM1:GD1a and GM1:GQ1b complexes were prominent pairings, many of these samples containing completely complex-dependent antibodies (i.e., without the complex, the antibody would not be identifiable as there was no reactivity above threshold for each individual ganglioside). Thus of the 98 (36.8%) GBS sera that bound GM1:GD1a complex, 25 were completely complex dependent. Of the 98 (36.8%) GBS sera that bound GM1:GQ1b complex, 27 were completely complex dependent (figure 2, E–F).

Antibody binding events were infrequent in the AMAN population for GA1 (37/102; 36.3%) and GD1a (14/102; 13.7%) when considered as single antigens, compared with other GBS populations in which anti-GD1a-positive AMAN cases are more prominent.^6^ However, the GA1:GD1a complex was a significant target in 36 (35.3%) AMAN samples, of which 10 were completely complex dependent (figure 2, B–D). Similarly, GM1:GD1a was a significant target in 57 (55.9%) AMAN samples, of which 14 were complex dependent. In this clinical population, both GA1 and GM1 therefore appear to enhance anti-GD1a antibody detection when the 2 glycolipids are in complex.

### Complex enhancement and complex inhibition.

In order to illustrate the enhancing and inhibiting effect of complexes, exemplary data are presented for GA1:GD1a (figure 2, B–D) and GM1:GQ1b (figure 2, E–G). Heterogeneous patterns are found, as expected. Thus there are 2 populations of anti-GA1:GD1a antibodies in GBS sera: (1) those in which binding intensities to GA1 or GD1a are attenuated by GA1:GD1a complex and (2) those in which binding is greatly enhanced by the complex, most usually in a completely complex-dependent fashion (i.e., no binding is present to either partner alone; figure 2D, green lines). Similarly, when considering GM1 and GQ1b, while some anti-GM1 and anti-GQ1b sera have attenuated binding intensities when in GM1:GQ1b complex, there is a significantly large group of sera in which major enhancement occurs in the presence of the complex (figure 2G, green lines).

### The added value and limitations of screening for heteromeric complexes.

To assess the overall impact of including glycolipid heteromeric complexes in the screening platform, we compared the number of GBS samples classified as positive (i.e., returning an intensity value >95th percentile of the combined controls) for single glycolipids and heteromeric complexes for the 10 frequently observed reactivities (figure 2A). A total of 131 of 266 GBS samples and 87 of 579 combined controls were positive for one or more single targets (sensitivity 49.2%, specificity 85.0%), whereas 155/266 GBS samples and 95/579 combined controls were positive for one or more heteromeric complexes (sensitivity 58.3%, specificity 83.6%). Therefore, by screening for heteromeric complexes, a gain of 9.1% in sensitivity (*p* = 0.0021) is achieved without a significant loss in specificity (1.4%; *p* = 0.445). Screening of GBS sera, irrespective of the clinical variant (AMAN, AIDP, or unclassified), against this discrete panel of 10 glycolipids (4 single and 6 complexes) resulted in an overall assay sensitivity of 64.3% and specificity of 77.1%.

**Figure 3:**
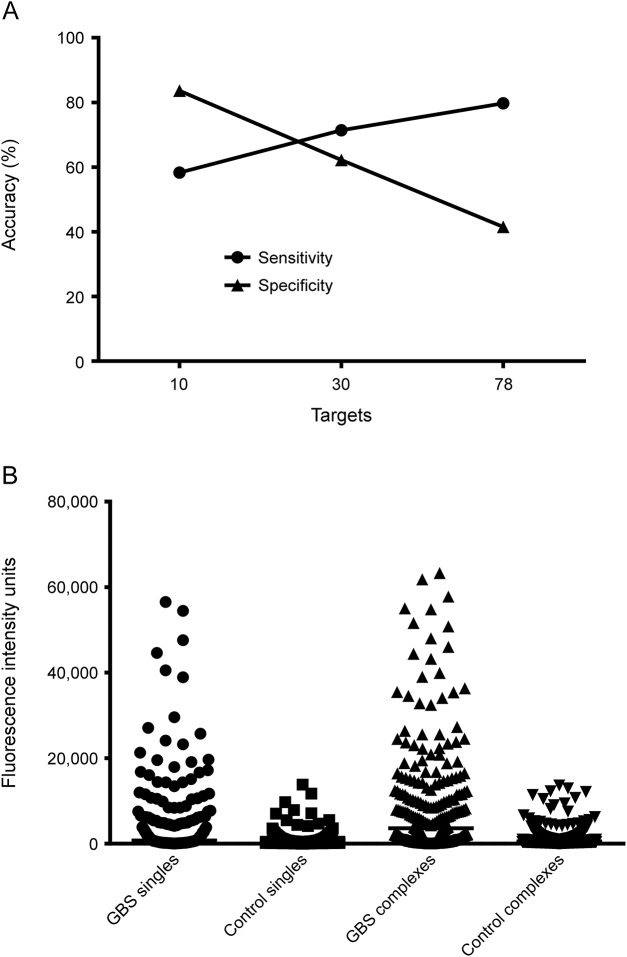
Sensitivity and specificity of microarray analysis in relation to target number (A) Comparison of overall assay accuracy (both sensitivity and specificity) when screening against an increasing number of target antigens. Assay specificity declines as the numbers of target antigens increases. Improved assay performance is achieved when a small panel of 10 targets is selected. (B) Dot plot compares the maximum binding intensity signal for each patient or control when comparing single and heteromeric complexes. GBS patient median arbitrary binding intensity for single targets (747.3 FIU) is significantly less than for heteromeric complex targets (3589.9 FIU; *p* < 0.0001), indicating that heteromeric complexes return higher signals than single antigens.

However, when examining a larger panel of targets, a gain in sensitivity is frequently offset by an equivalent or greater loss in specificity (figure 3A). For example, when examining the 30 targets with significantly different fluorescent intensity values (figure 1B), a gain of 30.4% in sensitivity (from 41.0% to 71.4%) is offset by a loss of 29% specificity (from 91.2% to 62.2%) when comparing single and complex positivity frequency. Similarly, when considering all 78 targets printed on the glycolipid arrays, there is a 14.7% gain in sensitivity (from 65.0% to 79.7%), while specificity drops 24.3% (from 65.8% to 41.5%) for single and complexes, respectively. These data show that when examining the data in its totality, the disease specificity of a very broad unbiased screen is expectedly poor, owing to the large number of controls whose sera contain at least one species of antiglycolipid antibody above the normal range (>95th percentile).

### Antiglycolipid antibodies associated with clinical variants.

Correlation between antiglycolipid antibody profiles and clinical features are presented in table and as e-Results. Having determined a panel of 10 glycolipid reactivities optimized for sensitivity and specificity for all patients with GBS irrespective of the clinical variant, next we sought to determine whether the presence of specific antibody reactivities would segregate with a clinical variant, or could be used to predict the clinical phenotype or outcome (see e-Results). Patients who had unclassified GBS variants (n = 105) were excluded from this analysis. Six targets were identified as being significant in the AIDP population when compared with the AMAN clinical variant. These included SGPG alone, as well as 2 complexes containing SGPG (SGPG:GQ1b and SGPG:Chol). In addition, 2 heteromeric complexes containing LM1 (LM1:Sulphatide and LM1:Chol) and CTH:Chol were significantly associated with AIDP (*p* < 0.05). While significant, each of these targets was present at low frequencies within the patient samples, and thus individually they had low diagnostic sensitivity (ranging from 10.5% to 14.0%), but with a high specificity both for combined controls (95.0%) and patients with AMAN (ranging from 96.0% to 98.0%, depending upon the target). When combined as a diagnostic panel of 6 antigen targets for AIDP, a sensitivity of 33.9% and a specificity of 87.3% and 83.4% (for AMAN and controls, respectively) was reached.

**Table:**
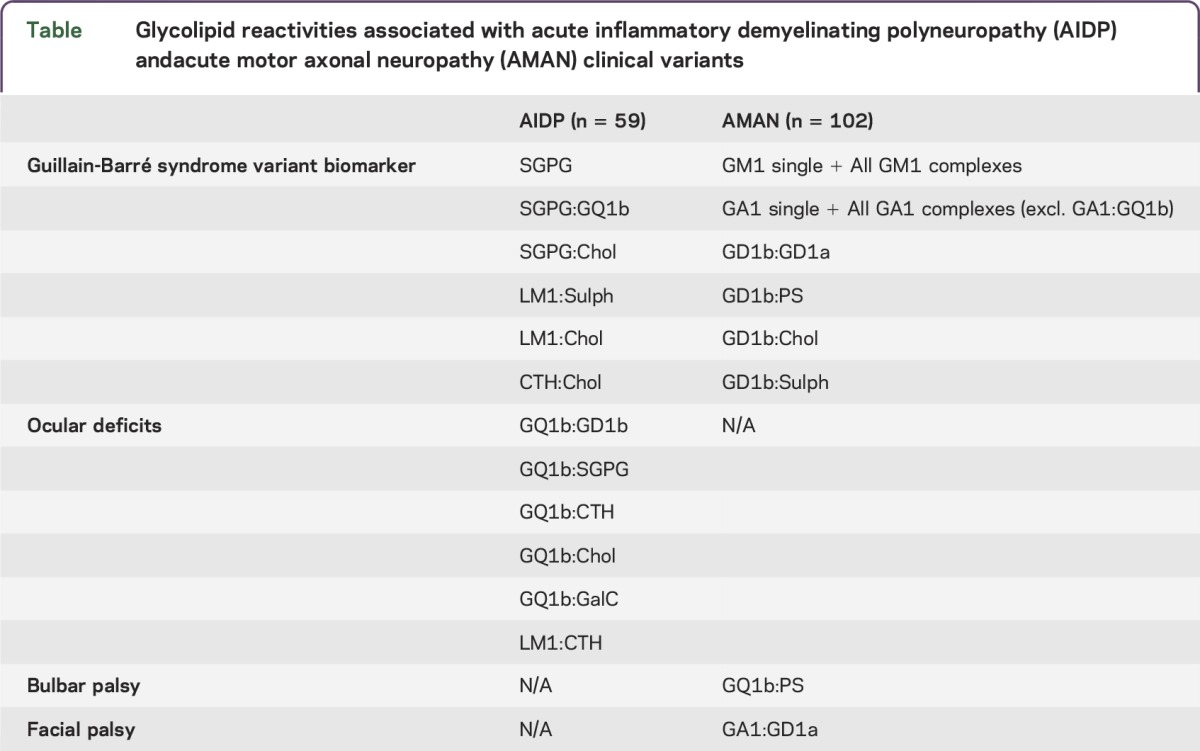
Glycolipid reactivities associated with acute inflammatory demyelinating polyneuropathy (AIDP) andacute motor axonal neuropathy (AMAN) clinical variants

**Figure 4:**
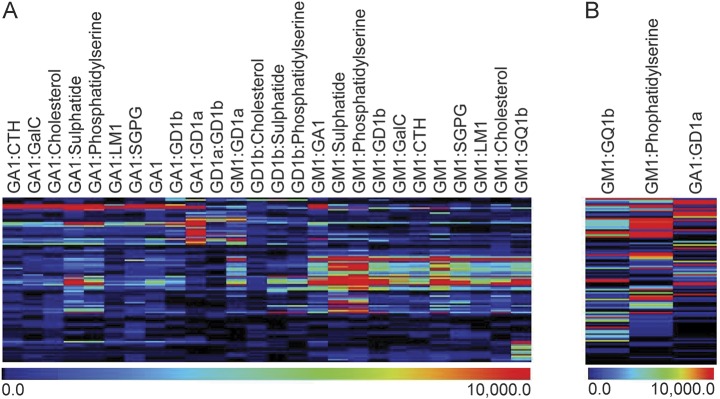
Heat map of antibody binding patterns in axonal vs demyelinating Guillain-Barré syndrome (GBS) cases (A) Heat map presentation of the 26 glycolipid targets significant for the acute motor axonal neuropathy variant of GBS compared with the demyelinating (acute inflammatory demyelinating polyneuropathy) clinical subtype. (B) Refining the screening targets to a panel of 3 heteromeric complexes (GM1:GQ1b, GM1:phosphatidylserine, and GA1:GD1a) results in a sensitivity of 78.4% and a specificity of 87.7% (for combined controls).

Twenty-six targets were identified as being significant (*p* < 0.05) in AMAN when compared with the AIDP population (figure 4A). These include GM1 alone plus GM1 in complex with all tested lipids (GM1:GA1, GM1:GD1a, GM1:GD1b, GM1:GQ1b, GM1:SGPG, GM1:LM1, GM1:CTH, GM1:PS, GM1:Chol, GM1:Sulphatide, GM1:GalC), GA1 alone plus GA1 in complex with all tested lipids, with the exception of GA1:GQ1b (GA1:GD1a, GA1:GD1b, GA1:SGPG, GA1:LM1, GA1:CTH, GA1:PS, GA1:Chol, GA1:Sulphatide, GA1:GalC), GD1b:GD1a, GD1b:PS, GD1b:Chol, and GD1b:Sulphatide. Of these, GM1:GD1a had the highest sensitivity (55.9%) and a high specificity for both combined controls (95.0%) and patients with AIDP (84.8%). When combined as a diagnostic panel of 26 antigen targets for AMAN, a sensitivity of 86.3% and a specificity of 64.4% and 45.8% (for combined controls and AIDP, respectively) was reached. As previously demonstrated, the specificity was reduced with increasing antigen targets; therefore, we sought to refine the number of antigen targets in order to optimize sensitivity and specificity. When selecting only 3 antigen targets (GM1:GQ1b, GM1:PS, and GA1:GD1a), a sensitivity of 78.4% and specificity of 87.7% and 61.0% (for combined control and AIDP, respectively) was achieved (figure 4B).

## DISCUSSION

This study applies technical developments in antiglycolipid antibody assay miniaturization to address the practical complexities of large-scale biomarker screening of clinical cohorts. By adapting the microarray printer to accommodate glycolipids, we were able to print arrays in a high throughput manner. One maximum capacity assay is capable of printing 320 individual arrays, on a total of 20 slides. Conducting an equivalent screen on this sample size and antigen repertoire in a conventional ELISA study would require 640 ELISA plates (96-well). Miniaturization of the glycolipid assay allows us to use 100-fold smaller volumes of patient serum, thus the total serum use by microarray is 2 μL to probe against 78 target antigens with duplicate measurement, compared with 200 μL of serum for a comparable study performed on ELISA. In addition, the use of fluorescently conjugated antibodies allows for multiplex screening, whereby IgG and immunoglobulin M can be detected simultaneously on each assay platform, a benefit not routinely available in ELISA readers.

In this study, a single electrophysiologic assessment was performed on 192 of the 266 patients in the GBS cohort. Of these patients, 53.1% of patients were classified as having an axonal variant of GBS, 30.2% of patients with AIDP, while the clinical variant of the remaining 16.7% of patients was unclassified. These diagnostic categorization frequencies are in line with a previously reported GBS cohort from Bangladesh.^7^ As found here, this previous report also identified a low incidence of patient serum containing anti-GD1a antibodies (14%), which was surprising given the high frequency of axonal GBS, in comparison with studies from elsewhere.^6,8–10^ However, probing Bangladesh sera against heteromeric complexes proved advantageous as it revealed populations of complex-dependent GA1:GD1a and GM1:GD1a antibodies, which were distinct from antibodies binding GD1a as a single antigen. Similarly, GM1:GQ1b complex-dependent antibody binding was identified as a distinct population in 11.9% of AMAN samples, when compared to GM1 and GQ1b alone. Using a refined panel of only 3 antiglycolipid targets, a high sensitivity and specificity was achieved for patients with AMAN in this cohort.

In addition to screening GBS patient samples, 579 combined control sera were examined. This screen identified variations in the baseline levels of antiganglioside antibodies reactivities for each of the targets. When performing conventional ELISA, the current gold standard method for antiganglioside antibody detection, a universal optical density threshold of positivity across all glycolipids, is widely used. In this geographically restricted study, we can see that in order to optimize both sensitivity and specificity of the assay, individual threshold must be calculated, in order to determine the normal range of these naturally occurring and GBS-independent anticarbohydrate antibodies.

In this study, we present data that identify the presence of both single and heteromeric glycolipid complex binding antibodies in this large GBS cohort. Initial biomarker screening employed a panel of 78 targets; however, the selective inclusion of a very limited panel of targets enabled optimization of both sensitivity and specificity due to significant target redundancy resulting from the presence of polyclonal antibodies or cross-reactivity of specific antibody species. When considering the overwhelming complexities of identifying diagnostically important glycolipid complexes by ELISA screening, the necessities of miniaturization are clearly evident for the initial analysis in order to find the markers that are most informative.

## Supplementary Material

Data Supplement

## GLOSSARY

AIDP: acute inflammatory demyelinating polyneuropathy
AMAN: acute motor axonal neuropathy
BSA: bovine serum albumin
FC: family controls
GBS: Guillain-Barré syndrome
IgG: immunoglobulin G
NC: neurologic disease controls
PBS: phosphate-buffered saline
ROC: receiver operating characteristic
