# Supplementary material for: Microarray screening of Guillain-Barré syndrome sera for antibodies to glycolipid complexes
Source: Neurol Neuroimmunol Neuroinflamm. 2016 Sep 28;3(6):e284. doi: 10.1212/NXI.0000000000000284 (PMC5055300; doi:10.1212/NXI.0000000000000284)
Supplement: Data Supplement [file supp_3_6_e284__index.html]

Data Supplement 

# Microarray screening of Guillain-Barré syndrome sera for antibodies to glycolipid complexes

## Data Supplement

**Files in this Data Supplement:**

- Data Supplement - Microsoft Word file
- Data Supplement - Microsoft Word file
