# Supplementary material for: Microarray screening of Guillain-Barré syndrome sera for antibodies to glycolipid complexes
Source: Neurol Neuroimmunol Neuroinflamm. 2016 Sep 28;3(6):e284. doi: 10.1212/NXI.0000000000000284 (PMC5055300; doi:10.1212/NXI.0000000000000284)
Supplement: Data Supplement [file supp_3.6.e284_Supplementary_methods_changes_highlighted_19.07.16.docx]

Supplementary Methods

Array platform fabrication

Array platforms were fabricated from sheets of low fluorescence PVDF membrane (Merck Millipore, Darmstadt, Germany) adhered to glass microscope slide using photo mount spray adhesive (3M). Excess adhesive and membrane were trimmed from the slide, which were then stored at room temperature, until required.

Clinical samples

Neurological control patients (NC; n=321) were diagnosed with the following; other neuropathy, 15 (4.7%); myopathy/myositis, 24 (7.5%); cerebrovascular accident, 88 (27.4%); motor neuron disease, 12 (3.7%); systemic lupus erythematosus, 3 (0.9%); intracranial space occupying lesion, 24 (7.5%); meningitis/encephalitis/transverse myelitis, 63 (19.6%); seizure/convulsion disorder, 14 (4.4%); myasthenia gravis, 8 (2.5%); other neurological disease, 70 (21.8%).

Family controls were selected based on shared place of habitation. The health status of family controls (FC; n=258), during the 2 weeks prior to study enrolment, were recorded as follows; healthy, 229 (88.8%); fever, 10 (3.9%); diarrhoea, 5 (1.9%); respiratory tract infection, 10 (3.9%), hypertension, 2 (0.8%); diabetes, 2 (0.8%).

Patient information and clinical presentation was recorded for each GBS patient at onset (age, sex, preceding symptoms, clinical subtype and cranial nerve involvement). In addition, clinical outcomes (sensory signs, ataxia, autonomic dysfunction, MRC sum score, functional disability score) were recorder for each GBS patient over a period of 1 years beginning at neurological onset, and followed up at 2 weeks, 1 month, 3 months, 6 months and 1 year intervals.

Glycolipids

The following lipids were printed; GM1, GA1, GD1a, GD1b, Phosphatidylserine (PS), Cholesterol, Sulphatide and Galactocerebrosides (GalC), all purchased from Sigma (Poole, UK), Ceramide trihexosides (CTH; Gb3) and GQ1b from Matreya (Pleasant Gap, USA) and SGPG and LM1 were kind gifts from Prof. R. Yu (Georgia Regents University, Augusta, USA). Working solutions of single glycolipids were prepared at 200µg/ml in methanol, from which heterodimeric complexes were prepared, having a total concentration of 200µg/ml (100µg/ml of each glycolipid) and stored at -20˚C until required. Lipid solutions were mixed in an ultra-sonic bath, for a minimum of 3 minutes, prior to application into the dispensing well, and immediately before uptake by the sciflexarrayer printing nozzle, thereby minimizing evaporation of the solvent.

Array program

Glycolipids were applied to the PVDF coated slides using a piezo-electric driven Sciflexarrayer S3, non-contact, microarray printer (Scienion, Berlin, Germany). Each print run can accommodate a maximum of 20 slides, each of which, containing 16 individual arrays (maximum 320 arrays printed per run). As standard, when utilising an x and y dot pitch of 300µm, each subarray contained a maximum of 400 spots. For optimised handling of methanol solvent, a type 1 coated piezo dispense capillary nozzle with a 90µm diameter opening (PDC90, Scienion) was employed under optimised voltage and pulse width, to produce an average single drop volume of 500pL. Each spot was composed of 2 drops per spot, resulting in 200pg of total glycolipid deposited per spot, with an average spot diameter of 200µm.The lipid printing program incorporated a 5µl air uptake step by the nozzle, prior to aspiration of 25µl of the lipid solution, in order to prevent admixing of the system liquid solution (filtered and degassed water) and lipid solution. The hydrophobic property of PVDF membrane enabled printed lipids to form non-covalent hydrophobic interactions with the array surface, upon rapid evaporation of the methanol solvent. This obviated the need to chemically alter the chemical structure of the glycolipid molecules, which enabled cis-interaction of the native molecule in their totality. Each sub-array contained fluorescence orientation spots in the 1^st^ and last row of the array, enabling the correct designation of positive spots. All glycolipid targets were printed in duplicate on each array and included methanol solvent which was printed as a negative control. During printing runs, a series of 3 methanol wash cycles were incorporated into the program between each application of glycolipid, to prevent cross contamination. Upon completion of printing, arrays were stored at 4˚C until required.

Sera screening

Non-specific serum binding was reduced by blocking arrays in 2% BSA/PBS, with gentle agitation, for 1 hour at room temperature. Arrays were then housed within a FAST frame containing a 16 well incubation chamber (Maine Manufacturing, Sanford, USA) and 100µl of each serum sample, diluted 1:50 in 1% BSA/PBS, was applied per well for 1 hour at 4˚C. Samples were aspirated from all wells using a multi-channel pipette followed by washing twice with 100µl of 1% BSA/PBS, prior to the removal of the array from the FAST frame, after which multiple arrays were washed twice en masse in 1% BSA/PSA, with gentle agitation, for 15 minutes at room temperature. The arrays are returned to the FAST frame containing incubation chamber and 100 µl of 2µg/ml (in1% BSA/PBS) of Alexafluor 647 conjugated goat anti-human IgG (Jackson ImmunoResearch), are applied per well for 1 hour at 4˚C. The arrays are then washed twice en masse in 1% BSA/PBS for 30 minutes, followed by twice in PBS for 5 minutes and a final 5 minutes wash in distilled water. Each serum samples was assayed in duplicate and screened twice in two independent assays.

Scanning and Analysis

Arrays were scanned and quantitated using a Perkin Elmer scanarray express instrument. Images were captured at 10µm resolution and a standardised PMT was employed for the duration of this study. Image analysis was carried out with ProScanArray Express Easy Quant software. Spots were defined as circular features with adaptive circle template applied for intensity measurements (diameter range 200-250µm). Each target spot was measured for median fluorescence intensity with local background median pixel intensity subtracted, and the mean value was calculated for each pair of duplicate spots. For negative values, these were reported as zero. Values obtained from repeat runs were averaged and used in all calculations. Data processing was performed with Microsoft Excel.

Statistical analysis

Analysis of intensity values was performed with aim to find significantly increased (p<0.05) binding intensities of single glycolipids and complexes in GBS as compared independently with both FC and NC groups. First, intensity values were offset by 1 and log2 transformed. Then, to accommodate large number of zero intensities, censored regression model (censReg R package) was applied. Finally, Bonferroni correction was applied to correct p-values for multiple testing.

Antibodies reactive to gangliosides and lipids are frequently observed in the normal population, albeit at lower binding intensities compared with inflammatory neuropathy patients, therefore the 95% percentile of the combined controls was selected as the threshold of positivity, for each target. As a result of employing this method, 29/579 (5%) of combined controls for any one target was considered positive (95% specificity). For comparison of proportionality data, as determined by the positivity threshold value, Fisher’s exact test was used, and for non-parametric rank testing, analysis was performed using Kruskal-Wallis with post-hoc analysis using Mann-Whitney test (GraphPad Prism software Inc., San Diego, USA). McNemar chi squared test was performed in MedCalc Version 14.12.0 software (MedCalc Software, Ostend, Belgium) for comparison of sensitivity and specificity of test results^1^. Receiver operator characteristic (ROC) analysis of the area under the curve (AUC) was employed using Hanley and McNeil methodology with 95% confidence intervals (MedCalc). In the event that a GBS patient’s clinical data was missing for a particular outcome measurement, the patient was omitted from the specific data analysis.

Raw intensity unit values were used to produce heat maps (MeV software; Dana-Farber cancer Institute, Boston, USA) presented in rainbow format (to facilitate visual display of the intensity measurements), which underwent hierarchical clustering using Pearson correlation for distance metric selection.

Microarray Assay Performance

In order to establish a measurement of the intra-assay precision, a single serum sample from one individual was divided into separate aliquots and then assayed on replicate microarray chips from the same print batch. Each aliquot was diluted 1:50 and 100µl of each was added per subarray to replicate slides from the same print run. Each aliquot was considered to be independent of all others for data analyses. For each glycolipid, single and complex, the mean signal, standard deviation and coefficient of variance (CV) across the 3 aliquots were calculated. The intra-assay CVs varied from 0% to 69.4%, with median CV=10.4% and average CV= 15.4%.

Reference List

1. Trajman A, Luiz RR. McNemar chi2 test revisited: comparing sensitivity and specificity of diagnostic examinations. Scandinavian journal of clinical and laboratory investigation 2008;68:77-80.
