# Supplementary material for: Microarray screening of Guillain-Barré syndrome sera for antibodies to glycolipid complexes
Source: Neurol Neuroimmunol Neuroinflamm. 2016 Sep 28;3(6):e284. doi: 10.1212/NXI.0000000000000284 (PMC5055300; doi:10.1212/NXI.0000000000000284)
Supplement: Data Supplement [file supp_3.6.e284_Supplementary_results_19.07.16.docx]

**Supplementary Results**

**Oculomotor deficits**

GBS patients with anti-GQ1b antibodies frequently have oculomotor deficits as found in the regional variant, Miller Fisher syndrome (MFS)^1-3^. In the current cohort, only one patient was diagnosed with MFS (herein categorised as ‘unclassified’) and anti-GQ1b antibodies were present which reacted against the single antigen and heteromeric complexes of GQ1b. Amongst the patient diagnosed with AMAN, no ophthalmoplegia was observed; however in 10.2% (6/59) of AIDP patients, ophthalmoplegia was recorded. In this group, 2/6 patients had anti-GQ1b antibodies, which did not reach significance when compared with AIDP patient without ophthalmoplegia (p=0.08). However, anti-GQ1b complex antibodies were statistically significant in the ophthalmoplegia group as follows; GQ1b:GD1b (p=0.01), GQ1b:SGPG (p=0.03), GQ1b:CTH (p=0.05), GQ1b:Chol (p=0.002) and GQ1b:GalC (p=0.01). In addition, antibodies against LM1:CTH (p=0.01) were also found at a higher frequency in this group.

**Bulbar and facial palsy**

Thirty seven of 102 (36.3 %) patients with AMAN had bulbar palsy at presentation. Of these patients, 6/37 (16.2%) had anti-GQ1b:PS complex antibodies compared with none of the AMAN patients without bulbar palsy (p=0.01).

Forty four of 59 (74.6%) AIDP patients also had bulbar palsy at presentation: amongst these patients there were no significant discriminatory markers. Facial palsy was present in 29/102 (28.4%) patients with AMAN. Of these 102 patients, 55.2% had anti- GA1:GD1a complex antibodies, compared with 20 of 73 (27.4%) AMAN patients without facial palsy (p=0.01). Amongst the 34 of 59 AIDP patients with facial palsy, there were no significant discriminatory markers.

**Sensory and autonomic features**

28 of 59 (47.5%) AIDP patients had sensory symptoms at presentation, compared with 10 of 102 (9.8%) AMAN patients (p<0.0001). Sensory ataxia was also more frequent in AIDP than AMAN patients at clinical presentation (p<0.0001). Closer analysis of the clinical data did not reveal any significant antibody targets associated with impairment of touch or positional and/or vibrational sense, or ataxia. There was also a higher incidence of autonomic dysfunction amongst AIDP patients at presentation compared with AMAN (p=0.03); again this was not associated with discernibly different anti-glycolipid antibodies.

**Disease severity defined by GBS disability score**

Each patient was assessed for functional disability (scored progressively worse from grade 1-5 with 0 = no functional disability and 6 = death) at entry, 2 weeks, 1 month, 3 months, 6 months and 1 year. The maximum severity score for each patient was determined, and examined according to clinical subtype diagnosis. Of the 266 patients diagnosed with GBS, 109 patients (41.0%) made a full recovery 1 year after onset, 117 patients (44.0%) had residual symptoms and signs, and 35 patients (13.2%) died within this observation period. Functional disability score data wasn’t available for the remaining 5 patients. In the AMAN group (n= 102 patients), 68.6% reached a peak functional disability score of 4 (chair bound or bedridden), and 9.8% reached a score of 5 (assisted ventilation). 30.4% of patients with AMAN had made a full recovery at the 1 year observation point, and 3 patients (2.9%) had died. In the AIDP group (n= 59 patients), 66.1% reached a peak functional disability score of 4, and 10.2% reached a score of 5. 64.4% of patients with AIDP had made a full recovery at the 1 year observation point and 4 patients died (6.8%). For neither AMAN nor AIDP clinical subtypes, no association was determined between disease severity and antibody target or binding intensity.

Reference list

1. Chiba A, Kusunoki S, Shimizu T, Kanazawa I. Serum IgG antibody to ganglioside GQ1b is a possible marker of Miller Fisher syndrome. Annals of neurology 1992;31:677-679.

2. Willison HJ, Veitch J, Paterson G, Kennedy PG. Miller Fisher syndrome is associated with serum antibodies to GQ1b ganglioside. Journal of neurology, neurosurgery, and psychiatry 1993;56:204-206.

3. Yuki N, Sato S, Tsuji S, Ohsawa T, Miyatake T. Frequent presence of anti-GQ1b antibody in Fisher's syndrome. Neurology 1993;43:414-417.
